# Strong cardiovascular prognostic implication of quantitative left atrial contractile function assessed by cardiac magnetic resonance imaging in patients with chronic hypertension

**DOI:** 10.1186/1532-429X-13-42

**Published:** 2011-08-15

**Authors:** Matthew Kaminski, Kevin Steel, Michael Jerosch-Herold, Maung Khin, Sui Tsang, Thomas Hauser, Raymond Y Kwong

**Affiliations:** 1 Cardiovascular Division, Department of Medicine, Brigham and Women's Hospital, Boston, Massachusetts, USA; 2 Department of Radiology, Brigham and Women's Hospital, Boston, Massachusetts, USA; 3 Cardiovascular Division, Beth Israel Deaconess Medical Center, Boston, Massachusetts, USA

## Abstract

**Background:**

Progressive left ventricular (LV) diastolic dysfunction due to hypertension (HTN) alters left atrial (LA) contractile function in a predictable manner. While increased LA size is a marker of LV diastolic dysfunction and has been shown to be predictive of adverse cardiovascular outcomes, the prognostic significance of altered LA contractile function is unknown.

**Methods:**

A consecutive group of patients with chronic hypertension but without significant valvular disease or prior MI underwent clinically-indicated CMR for assessment of left ventricular (LV) function, myocardial ischemia, or viability. Calculation of LA volumes used in determining LA emptying functions was performed using the biplane area-length method.

**Results:**

Two-hundred and ten patients were included in this study. During a median follow-up of 19 months, 48 patients experienced major adverse cardiac events (MACE), including 24 deaths. Decreased LA contractile function (LAEF_Contractile_) demonstrated strong unadjusted associations with patient mortality, non-fatal events, and all MACE. For every 10% reduction of LAEF_Contractile_, unadjusted hazards to MACE, all-cause mortality, and non-fatal events increased by 1.8, 1.5, and 1.4-folds, respectively. In addition, preservation of the proportional contribution from LA contraction to total diastolic filling (Contractile/Total ratio) was strongly associated with lower MACE and patient mortality. By multivariable analyses, LAEF_Contractile _was the strongest predictor in each of the best overall models of MACE, all-cause mortality, and non-fatal events. Even after adjustment for age, gender, left atrial volume, and LVEF, LAEF_Contractile _maintained strong independent associations with MACE (p < 0.0004), all-cause mortality (p < 0.0004), and non-fatal events (p < 0.0004).

**Conclusions:**

In hypertensive patients at risk for left ventricular diastolic dysfunction, a decreased contribution of LA contractile function to ventricular filling during diastole is strongly predictive of adverse cardiac events and death.

## Background

Left Ventricular (LV) diastolic dysfunction as a consequence of chronic hypertension is a prevalent condition associated with significant morbidity and mortality. Current strong prognostic markers that reflect diastolic dysfunction remain limited, but their identification may improve treatment planning and monitoring of patients with chronic hypertension. LA size reflects the duration and severity of exposure to increased diastolic filling pressures in the LV and is relatively load-independent. As LV diastolic impairment progresses, effective diastolic filling becomes increasingly dependent on LA contractile function until the LA contractile reserve can no longer meet the demand of elevated diastolic ventricular pressure[1-3]. While evidence exists that LA enlargement is a strong predictor of adverse cardiovascular outcomes in selected populations, there is little data regarding the prognostic implication of altered left atrial contractile function in patients with chronic hypertension at risk of diastolic dysfunction. While ‘ejection fraction’ is the phrase used for reduction of ventricular cavity volume, ‘emptying function’ is more appropriate for reductions of atrial volume as the atria lack inflow valves and empty passively as well as actively. Cardiac magnetic resonance imaging (CMR) can quantify left atrial volume and emptying functions with sufficient temporal and spatial resolution and excellent reproducibility. Accordingly, this study aims to test the hypothesis that altered LA contractile emptying functions as measured by cine CMR can provide strong prognostic information in patients with chronic hypertension, beyond left atrial volume and other known risk predictors in this population.

## Methods

### Patient Population

We studied a consecutive series of patients with history of chronic hypertension medically treated for at least 6 months who were referred to undergo cardiac magnetic resonance imaging (CMR) for clinical purposes. Patients were referred either for a) evaluation for myocardial ischemia with stress CMR or b) assessment of regional and global left ventricular function and myocardial mass. Patients with any of the following were excluded: a) any evidence of myocardial infarction by history, medical record, or abnormal cardiac enzymes, b) any significant aortic or mitral valvular dysfunction (moderate or severe dysfunction by qualitative echocardiographic grading), and c) confirmed (by biopsy) myocarditis, infiltrative cardiomyopathy (including cardiac hemochromatosis, amyloidosis, or sarcoidosis), or pericardial disease. Other exclusion criteria included concurrent unstable angina, NYHA class IV heart failure, hemodynamic instability, claustrophobia precluding CMR, and metallic hazards. Patients with patterns of late gadolinium enhancement consistent with infiltrative cardiomyopathy or myocarditis were also excluded. The institutional ethics committee of the Brigham and Women's Hospital (Partners Healthcare system) approved the clinical follow-up activities of the study.

### Clinical Evaluation

All patients underwent a detailed history immediately before the CMR. Hypertension, hypercholesterolemia, diabetes, and family history of premature CAD were defined by published criteria[4-7]. Significant smoking was defined by >10 pack-years of tobacco use. History of CAD included documented >70% stenosis on angiography or history of coronary revascularization prior to CMR study.

### CMR Imaging

All patients were studied supine in a 1.5T CMR system (Signa^® ^CV/*i*, GE Healthcare, USA) with a 4-element or 8-element phased-array surface coil. CMR study consisted of cine SSFP imaging (TR/TE 3.4/1.2ms, in-plane spatial resolution 1.6 × 2 mm, matrix 192 × 160) of LV function and late gadolinium enhancement imaging (TR/TE 4.8/1.3ms, TI 200-300ms) for myocardial scar. All images were acquired using retrospective ECG gating and breath-holding. Cine and late enhancement imaging were obtained in 8-14 matching short-axis (8 mm thick with 0 mm spacing) and 3 radial long-axis planes. The 3 radial long-axis planes were prescribed at 60 degrees apart. Typical view per segment during a cine SSFP acquisition was 12 yielding a temporal resolution of approximately 45 ms and maintaining a breath-hold of approximately 10-12 seconds for each slice location. A previously described segmented inversion-recovery pulse sequence for late enhancement imaging was used[8] starting at 15 minutes after cumulative 0.15 mmol/Kg dose of gadolinium-DPTA. A single reader categorized late gadolinium enhancement as either typical infarction (involving the subendocardium) or atypical (subepicardial, patchy midwall or diffuse circumferential subendocardial pattern).

### CMR Quantitative Analysis of Left Atrial Volume and Emptying Function

**Figure 1:**
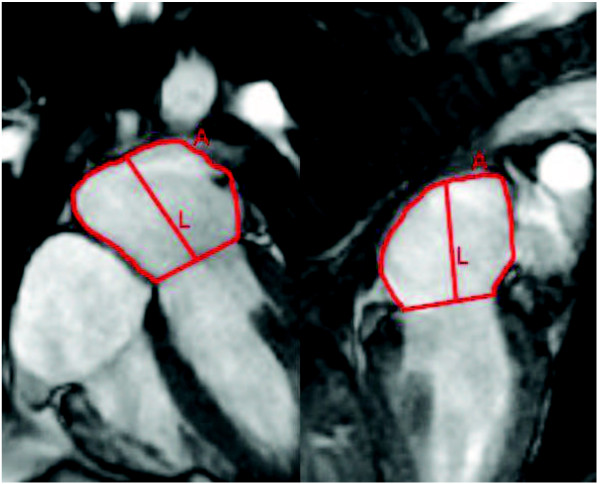
**Measurement of left atrial area (A) and length (L) in 4-chamber and 2-chamber views used in the calculation of the left atrial volume indices (LAV) across different phases of the cardiac cycle**.

Quantitative Analysis of the left atrium is illustrated in Figure 1. To measure the left atrial dimensions, manual tracings were made of the left atrial area and long axis in the radial 2-chamber and 4-chamber views. For each radial view, tracings were performed at three phases: maximal LA volume just before mitral valve opening, minimal LA volume at mitral valve closure, and immediately prior to atrial contraction. At each phase, LA volume is calculated by the previously validated biplane area-length method[9] as follows: LA volume (ml) = 0.85*A_2C_*A_4C_/L, where A_2C _and A_4C _are the LA areas on the 2-chamber and 4-chamber views, respectively, and L is the shorter long-axis length of the LA from either the 2-chamber or the 4-chamber views (Figure 1). Consistent with the recommendation of published reports[10], all LA volumes were normalized to the patient's body surface area in subsequent analyses. Total LA emptying volume was calculated as the difference between maximum (LAV_max_) and minimum LA volumes (LAV_min_). Total LA emptying volume was divided into LA passive emptying volume (V_Passive_) and LA contractile volume (V_Contractile_), in which V_Passive _was calculated as the difference between LAV_max _and the LA volume preceding atrial contraction (LAV_ac_) and V_Contractile _was calculated as the difference between LAV_ac _and LAV_min_. LA total, passive, and active emptying functions (LAEF_Total_, LAEF_Passive_, LAEF_Contractile_) were calculated according to the following formulas:

In additon, we calculate the proportional contribution of LA contraction during diastole by calculating the following parameters:

While left atrial dimension (in millimeters) was made available to the attending physicians on the day of the CMR, all other quantitative measurements of the left atrium were not reported as part of the routine clinical care.

### CMR Quantitative Analysis of LV Function and Definition of Myocardial Ischemia

All images were analyzed with specialized software (CineTool 2.80, General Electric Healthcare). We graded segmental systolic wall motion as normal or abnormal in each study and also graded segmental wall motion using a 4-point scale (1 = normokinesia, 2 = hypokinesia, 3 = akinesia, and 4 = dyskinesia) according to the standard 17-segment ACC/AHA nomenclature[11]. We interpreted late gadolinium myocardial enhancement (LGE) as present or absent. Details of segmental wall motion and LGE grading were as previously reported[12]. We manually traced epicardial and endocardial borders of matching short-axis cine locations at end systole and end-diastole to determine the LV ejection fraction (LVEF), end-diastolic volume index (LVEDVI), end-systolic volume index (LVESVI), and the LV myocardial mass (end-diastole only)[13,14]. LVEF was measured by standard Simpson's Rule[14]. Presence of ischemia during dobutamine stress was defined by standard criteria of worsening regional wall motion by at least 1 grade, matching on short and long-axis cine, as published in prior reports[15]. Presence of ischemia during adenosine stress perfusion was according to prior publication, defined by existence of perfusion defect without infarction by LGE imaging[16].

### ECG Interpretation

Resting 12-lead ECGs were obtained at a median of 1 day (interquartile range: 0-7 days) from CMR. We confirmed that no cardiac event or revascularization occurred between the collection of ECG and the CMR study. ECG interpretation was first performed by computer analysis followed by visual over-reading by a single reader blinded to the CMR results and the clinical outcome. ECG left atrial enlargement was defined by a terminal negative P-wave duration of > 40 ms and depth ≥ 1 mm measured on lead V1. We used the Sokolow-Lyon index to indicate LV hypertrophy on ECG[17].

### Follow-Up

At least 6 months following the CMR, clinical information was obtained from patient telephone interviews, contacting patients' physicians, and hospital records. A standard questionnaire was used during telephone interview. Survival was obtained from the National Social Security Death Index in patients lost on first contact[18]. Major adverse cardiac events (MACE) included any of the following: 1) all-cause mortality, 2) new acute myocardial infarction, 3) unstable angina requiring hospitalization, and 4) development or progression of heart failure requiring hospitalization. New acute myocardial infarction was defined by significant elevation of serum troponins consistent with myocardial injury. Unstable angina was defined by new chest pain hospitalization without non-cardiac origin of chest pain, and with either angiographic coronary stenosis of ≥70% or ischemia on noninvasive imaging. Heart failure was defined by a need for hospitalization for new or worsening symptoms of heart failure as determined by the patient's cardiologist or primary internist. When a patient experienced >1 MACE, the first event was chosen. When ≥2 MACE occurred simultaneously, the worse event was chosen (death>MI>unstable angina>congestive heart failure).

### Statistical Analysis

Baseline demographic differences, classified by the median LAEF_Contractile_, were compared by Student's t-test or Fisher's exact test. Kaplan-Meier distributions for MACE, all-cause mortality, and nonfatal events were stratified by the median value of LAEF_Contractile _and were compared by log-rank tests. We fitted Cox proportional-hazards models to estimate the likelihood ratio chi-square (LRχ^2^) and the unadjusted hazard ratios (HR) of all the variables. We also assessed the univariable association of the proportional contribution of LA contraction during diastole (Contractile/Passive and Passive/Total ratios) with MACE and other other events. To determine the set of variables that were most strongly associated with LAEF_Contractile_, we performed linear regression using LAEF_Contractile _as a continuous dependent variable, with all variables in Table 1 treated as independent variables. The prognostic association of LA size measurements and mechanical function estimates were also determined using similar analyses for all-cause mortality and non-fatal events specifically. We tested the interobserver agreement of LAEF_Contractile _by Spearman correlation.

**Table 1:** Demographic Characteristics of the Study Cohort

	All Patients (n = 210)	LA contractile function ≥ median (n = 96)	LA contractile function < median (n = 114)	P-value
Clinical Characteristics				
Age in years	52 ± 16	52 ± 16	51 ± 15	NS
Female Gender (%)	87 (41)	42 (44)	45 (39)	NS
Resting Heart Rate	71 ± 15	71 ± 15	71 ± 15	NS
Hx. of Diabetes (%)	71 (34)	32 (33)	39 (34)	NS
Hx. of Hypercholesterolemia (%)	138 (65)	60 (63)	78 (68)	NS
Heavy Tobacco Use (%)	56 (27)	24 (25)	32 (28)	NS
Hx of PAD (%)	21 (10)	6 (6)	15 (13)	NS
Hx. of Percutaneous Coronary Intervention (%)	25 (12)	12 (13)	13 (11)	NS
Hx. of Cardiac Bypass Surgery (%)	21 (10)	6 (6)	15 (13)	NS
Hx. of Angiographic Coronary Stenosis Before CMR (%)	45 (21)	22 (23)	23 (20)	NS
Any Hx. of CAD before CMR (%)	41 (20)	17 (18)	24 (21)	NS
Resting SBP	145 ± 25	148 ± 25	142 ± 24	NS
Resting DBP	75 ± 13	76 ± 12	75 ± 15	NS
Medication				
Beta-blocker (%)	136 (65)	55 (57)	81 (71)	0.04
Calcium Blocker (%)	52 (25)	26 (27)	26 (23)	NS
Angiotensin-converting enzyme inhibitor (%)	103 (49)	51 (53)	52 (46)	NS
Aspirin (%)	116 (55)	51 (53)	65 (57)	NS
Nitrates (%)	23 (11)	9 (9)	14 (12)	NS
Digoxin (%)	4 (2)	1 (1)	3 (3)	NS
Rest Electrocardiogram^£^				
Left Atrial Enlargement on ECG (%)	24 (11)	13 (14)	10 (9)	NS
Left Ventricular Hypertrophy on ECG	17 (8)	7 (7)	10 (9)	NS
QRS duration (ms)	100 ± 25	97 ± 20	102 ± 27	NS
Left bundle branch block (%)	19 (9)	9 (9)	10 (9)	NS
Right bundle branch block (%)	16 (8)	3 (3)	13 (11)	0.05
QTc interval	439 ± 39	433 ± 36	443 ± 40	0.09
Significant Q-waves by Minnesota Code Criteria	19 (9)	12 (12)	7 (6)	NS
CMR				
Ao root dimen (mm)	28 ± 5	28 ± 4	28 ± 5	NS
LVEF (%)	58 ± 13	61 ± 10	55 ± 15	<0.01
LV mass (grams)	142 ± 50	139 ± 50	145 ± 51	NS
LVEDD (mm)	54 ± 8	53 ± 8	55 ± 8	NS
LVEDV index (ml/m^2^)	164 ± 59	160 ± 54	167 ± 63	NS
LVESV index (ml/m^2^)	73 ± 50	65 ± 41	79 ± 57	0.04
LA Volume index - end-systole (ml/m^2^)	51 ± 20	45 ± 16	57 ± 21	<0.0001
LA Volume index - before atrial contraction (ml/m^2^)	41 ± 18	72 ± 31	88 ± 38	0.001
LA Volume index - end-diastole (ml/m^2^)	30 ± 19	21 ± 9	39 ± 22	<0.0001
LAEF_Passive _(%)	19 ± 12	20 ± 12	18 ± 12	NS
LAEF_Contractile _(%)	32 ± 15	42 ± 7	21 ± 13	<0.0001
LAEF_Total _(%)	44 ± 16	54 ± 9	34 ± 15	<0.0001
Contractile/Passive Ratio	3.8 ± 19	5.1 ± 25	2.4 ± 8	<0.0001
Contractile/Total Ratio	0.57 ± 0.29	0.64 ± 0.18	0.50 ± 0.37	<0.0001

We performed 2 separate multivariable approaches to analyze the predictive value of CMR variables for MACE and all-cause mortality. In the first approach, we sought to determine the strongest set of variables that were associated with MACE, all-cause mortality, and non-fatal events, respectively, in this study cohort. We used a stepwise forward selection strategy and considered all clinical, ECG, or left ventricular variables as listed in Table 2. A p-value of 0.1 was used as criteria for variable inclusion or exclusion. In the second approach, we aimed to determine the prognostic association, if any, of LAEF_Contractile _after adjustment to well-known risk predictors. Therefore, we sought to determine if LAEF_Contractile _remained a significant predictor after adjustment to patient age, gender, minimal left atrial volume, and left ventricular ejection fraction. The validity of the proportional-hazards assumptions of this final model for MACE was again tested and was valid for all the variables in this model. All analyses were performed with SAS 9.1 (SAS Institute, Cary, N.C.) for Windows.

**Table 2:** Univariable Association of Variables with All MACE, All-Cause Mortality, and Non-fatal Events

	All MACE (N = 48)			All-Cause Mortality (N = 21)			Non-Fatal Events (N = 27)		
	LRχ^2^	HR (95% CI)	P-value	LRχ^2^	HR (95% CI)	P-value	LRχ^2^	HR	P-value
Age (years)	0.41	1.01 (0.99-1.03)	NS	0.51	1.01 (0.98-1.04)	NS	0.02	1.00 (0.97-1.03)	NS
Female Gender	2.12	1.63 (0.84-3.15)	NS	4.65	2.72 (1.10-6.73)	0.03	0.01	1.04 (0.41-2.63)	NS
Body Mass Index (m/kg^2^)	1.62	0.96 (0.91-1.02)	NS	0.19	0.98 (0.91-1.06)	NS	2.19	0.94 (0.86-1.02)	NS
Hx. Percutaneous Coronary Intervention	3.21	2.07 (0.93-4.56)	0.07	0.54	1.51 (0.51-4.48)	NS	1.97	2.25 (0.73-6.98)	NS
Hx. Coronary Bypass Surgery	3.69	2.25 (0.96-5.13)	0.05	0.23	1.35 (0.40-4.58)	NS	3.44	2.87 (0.94-8.74)	0.06
Diabetes	0.49	1.27 (0.65-2.47)	NS	0.01	1.00 (0.41-2.42)	NS	0.07	1.14 (0.44-2.96)	NS
Hypercholesterolemia	0.10	1.12 (0.54-2.34)	NS	0.82	0.67 (0.28-1.61)	NS	1.53	2.19 (0.63-7.59)	NS
Hx. Heavy Smoking	0.60	1.31 (0.66-2.57)	NS	0.17	0.82 (0.31-2.12)	NS	1.09	1.65 (0.64-4.22)	NS
Family Hx. CAD	1.00	0.66 (0.29-1.49)	NS	0.33	0.77 (0.32-1.85)	NS	0.47	0.67 (0.21-2.11)	NS
Medications									
Beta-blocker Use	4.68	2.63 (1.10-6.33)	0.03	4.27	4.65 (1.08-19.96)	0.04	1.08	1.80 (0.59-5.49)	NS
Calcium Channel Blocker Use	1.08	1.44 (0.72-2.89)	NS	0.01	0.95 (0.35-2.59)	NS	1.66	1.87 (0.72-4.83)	NS
ACE Inhibitor Use	0.05	0.93 (0.48-1.79)	NS	0.01	0.98 (0.42-2.31)	NS	0.74	0.66 (0.25-1.70)	NS
Aspirin Use	0.97	1.42 (0.71-2.83)	NS	0.04	0.92 (0.39-2.18)	NS	1.43	1.88 (0.67-5.27)	NS
ECG Variables									
History of Atrial Fibrillation on ECG	2.17	2.22 (0.77-6.43)	NS	1.22	2.30 (0.53-10.05)	NS	0.66	1.87 (0.41-8.46)	NS
Left Atrial Enlargement on ECG	0.01	1.04 (0.36-2.96)	NS	0.62	0.45 (0.06-3.45)	NS	0.61	1.65 (0.47-5.82)	NS
LVH Voltage and Strain	1.92	1.97 (0.75-5.14)	NS	**	**	NS	7.58	4.43 (1.54-12.77)	<0.01
Significant Q Waves	0.64	1.53 (0.54-4.39)	NS	3.73	3.00 (0.98-19.12)	0.05	**	**	**
QRS Interval > 120 ms	0.39	0.72 (0.25-2.04)	NS	0.29	0.67 (0.15-2.91)	NS	0.01	1.08 (0.31-3.77)	NS
Left Bundle Branch Block	1.21	1.71 (0.66-4.44)	NS	0.05	1.18 (0.27-5.15)	NS	3.31	2.84 (0.92-8.74)	0.07
Right Bundle Branch Block	0.23	0.70 (0.17-2.94)	NS	0.28	1.49 (0.34-6.49)	NS	**	**	**
Significant ST Changes	1.61	1.72 (0.74-3.98)	NS	0.06	0.83 (0.19-3.62)	NS	3.05	2.55 (0.89-7.32)	0.08
Significant T Changes	0.03	1.08 (0.47-2.49)	NS	0.10	1.19 (0.39-3.62)	NS	0.07	0.84 (0.24-2.94)	NS
Prolonged corrected QT Interval	5.15	2.23 (1.12-4.47)	0.02	1.42	1.76 (0.70-4.43)	NS	4.58	2.99 (1.10-8.14)	0.03
CMR Variables of the LV									
LVEDD (mm)	0.70	1.02 (0.98-1.06)	NS	2.93	0.95 (0.90-1.01)	0.09	8.32	1.08 (1.02-1.14)	<0.01
LVEF (per 10%)	2.59	0.84 (0.68-1.04)	NS	0.04	1.03 (0.75-1.42)	NS	5.3	0.71 (0.54-0.95)	0.02
LVEDVI (per 10 ml/m^2^)	0.52	1.02 (0.97-1.07)	NS	4.06	0.89 (0.80-1.00)	NS	6.97	1.08 (1.02-1.13)	<0.01
LVESVI (per 10 ml/m^2^)	3.66	1.05 (1.00-1.10)	0.06	1.00	0.94 (0.82-1.07)	NS	10.35	1.09 (1.03-1.14)	0.001
LV Mass (gram)	0.01	1.00 (0.99-1.01)	NS	3.26	0.99 (0.98-1.00)	0.07	1.71	1.00 (1.00-1.01)	NS
Segmental Wall Motion Abnormality	1.18	1.49 (0.73-3.06)	NS	0.66	1.49 (0.57-3.93)	NS	0.17	1.24 (0.44-3.49)	NS
Abnormal LV LGE	6.33	2.47 (1.22-5.00)	NS	2.12	2.08 (0.78-5.59)	NS	3.91	2.69 (1.01-7.18)	0.05
CMR Variables of the LA									
Anteroposterior LA Dimension (mm)	4.64	1.05 (1.00-1.09)	0.03	1.01	1.03 (0.97-1.10)	NS	4.45	1.06 (1.00-1.12)	0.03
LAV_max _(ml/m^2^)	2.50	1.01 (1.00-1.03)	NS	1.60	1.01 (0.99-1.04)	NS	2.94	1.02 (1.00-1.04)	0.09
LAV_ac _(ml/m^2^)	3.16	1.02 (1.00-1.03)	0.08	2.79	1.02 (1.00-1.04)	0.10	2.99	1.02 (1.00-1.04)	0.08
LAV_min _(ml/m^2^)	6.28	1.02 (1.00-1.03)	0.01	6.48	1.02 (1.00-1.03)	0.01	3.84	1.02 (1.00-1.03)	0.05
LAEF_Passive_(per 10% reduction)	0.47	1.12 (0.81-1.57)	NS	0.39	1.16 (0.73-1.86)	NS	0.46	1.17 (0.74-1.86)	NS
LAEF_Contractile_(per 10% reduction)	**18.84**	**1.75 (1.36-2.24)**	**<0.0001**	**14.17**	**1.46 (1.20-1.77)**	**<0.001**	**6.94**	**1.35 (1.08-1.70)**	**<0.01**
LAEF_Total _(per 10% reduction)	10.91	1.45 (1.16-1.82)	<0.001	10.80	1.38 (1.14-1.68)	0.001	5.05	1.29 (1.03-1.60)	0.02
Contractile/Passive ratio	**10.64**	**0.94 (0.91-0.98)**	**<0.001**	**1.47**	**0.88 (0.72-1.08)**	**NS**	**11.9**	**0.93 (0.90-0.97)**	**<0.001**
Contractile/Total Ratio	**10.48**	**0.39 (0.24-0.64)**	**<0.001**	**8.75**	**0.21 (0.08-0.59)**	**<0.01**	**1.21**	**0.48 (0.13-1.77)**	**NS**

* Too Few Events for Analysis

## Results

### Baseline Characteristics

From a consecutive series of 225 patients with a history of chronic hypertension, 3 (1%) had unsuccessful CMR due to large body habitus or technical problem. Seven patients (3%) had moderate or severe aortic or mitral valvular dysfunction and were excluded from the study. Five patients were lost to follow-up but were reported alive. The remaining 210 (123 male, mean age 52 ± 16 years) formed the study cohort and were followed for a median 19 months (range 6-50 months). Patients were referred to undergo stress CMR for evaluation of ischemia (n = 106) or for assessment of left ventricular function (n = 104). Table 1 illustrates the demographical and ECG features of the entire study cohort and also data stratified by the median value of LAEF_Contractile _(33%). Baseline average left ventricular ejection fraction at rest was low normal at 58 ± 13%. During the study period, 53 patients were referred to undergo coronary angiography at a median 34 days (interquartile range 1-12 days) from CMR study. Among them 38 patients (72%) were found to have significant (>70%) luminal stenosis. LAEF_Contractile _dichotomized by its median level did not show correlation to the presence of angiographic coronary stenosis, history of percutaneous coronary intervention, or history of cardiac bypass surgery. Patients with LAEF_Contractile _below median value demonstrated a larger LA antero-posterior dimension (42 ± 9.1 mm vs. 38 ± 6.2 mm, P < 0.01), lower left ventricular ejection fraction (55 ± 15% vs. 61 ± 10%, P < 0.01), and higher left ventricular end-systolic volume index (79 ± 57 ml/m^2 ^vs. 65 ± 41 ml/m^2^, P < 0.05). There was high inter-observer correlation in quantifying LA volume (kappa statistic 0.83) across the three time phases of the cardiac cycle.

### Cardiovascular Outcome

At a median follow-up of 19 months (range 6 to 47 months), 48 MACE occurred. Among them were 21 cases of death, 3 acute myocardial infarction, 12 cases of hospitalization for unstable angina, and 12 cases of congestive heart failure requiring hospitalization.

### Relationship of Left Atrial Enlargement by ECG, LV Mass, and Left Atrial Volume and Mechanical Functions

Twenty patients (10%) demonstrated left atrial enlargement on ECG. The presence of left atrial enlargement by ECG was only weakly associated with a higher than median LAV_min _(P = 0.045) and did not demonstrate significant correlation with above median LAV_max_. Left atrial enlargement by ECG demonstrated poor sensitivity (16%) but excellent specificity (93%) in identifying patients with above median LAV_min_. Left atrial enlargement by ECG could not differentiate above or below median level of LAEF_Contractile_. While LV mass (g) is slightly higher in patients with above median LAV_min _(134 ± 44 vs. 152 ± 55, P = 0.01), it was not significantly different among patients with above or below median level of LAEF_Contractile_. Left ventricular ejection fraction was negatively correlated with LAV_min _(r = -0.19, P = 0.008) and positively correlated with LAEF_Contractile _(r = 0.15, p = 0.04). By multivariable linear regression, a history of atrial fibrillation on ECG, LAV_min_, and left ventricular ejection fraction were most strongly predictive of LAEF_Contractile_.

### Prognostic Association of Left Atrial Volume and Left Atrial Mechanical Function of the Cohort

**Figure 2:**
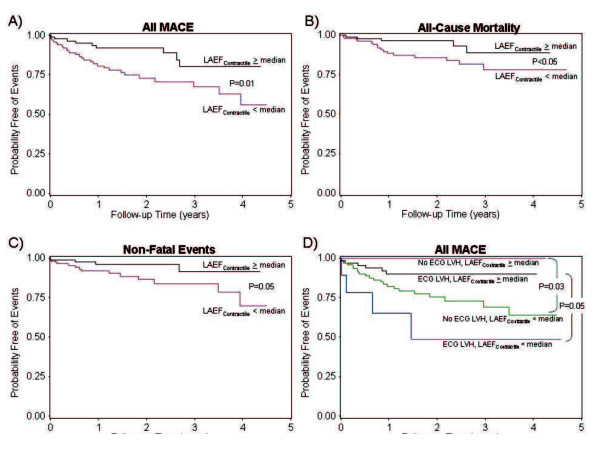
**Kaplan-Meier curves illustrating the time-to-event distributions of MACE, all-cause mortality, and non-fatal events of the study cohort, stratified by ≥ or < median LAEF_Contractile _(Figure 2A-C)**. In addition, median LAEF_Contractile _provided incremental prognostic association with MACE beyond ECG evidence of left ventricular hypertrophy (ECG LVH) (Figure 2D).

Univariable association of all variables with MACE, all-cause mortality, and non-fatal events is listed in Table 2. In the current study cohort, presence of LVH on ECG did not demonstrate significant association with MACE or mortality but was associated with non-fatal cardiac events (HR 4.43, 95% CI 1.54-12.77, P < 0.01). LV mass measurement did not demonstrate significant association with MACE. Decreasing left ventricular ejection fraction, increasing LAV_min _or LAV_max_, and presence of late gadolinium myocardial enhancement were all associated with non-fatal events but did not achieved significant association with MACE or mortality. LAV_min _(in ml/m^2^), LAEF_Contractile_, and LAEF_Total _demonstrated strong association with patient mortality, non-fatal events, and all MACE. Figure 2 illustrates the Kaplan-Meier curves demonstrating the time-to-event distributions for MACE, all-cause mortality, and non-fatal events, stratified by the median level of LAEF_Contractile _(Figure 2A-C). The prognostic association of LAEF_Contractile _with all MACE maintained its significance regardless of the presence or absence of ECG finding of LVH (Figure 2D). For every 10% reduction of LAEF_Contractile_, unadjusted hazards to MACE, all-cause mortality, and non-fatal events increased by 1.8, 1.5, and 1.4-folds, respectively. By similar argument, preservation of the proportional contribution of diastolic filling by atrial contractile, as quantified by the Contractile/Passive or Contractile/Total ratio, demonstrated strong association with favorable outcome to MACE (unadjusted HR 0.94 and 0.39, respectively, both P = 0.001), with a preserved Contractile/Total ratio portended to lower likelihood to mortality (HR to mortality 0.21, P = 0.003). On the contrary, LA antero-posterior dimension only demonstrated a weak unadjusted prognostic association with non-fatal events but not with all-cause mortality (Table 2).

**Figure 3:**
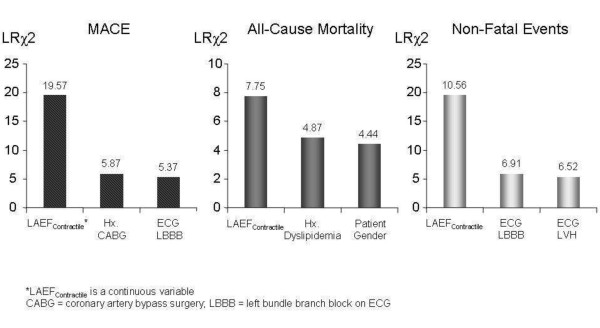
**Multivariable Best Overall Models for MACE, All-Cause Mortality, and Non-Fatal Events**. The respective LRχ^2 ^of the predictors in each model is illustrated by the number on top of each column.

In the first multivariable approach, stepwise forward selection strategy demonstrated that LAEF_Contractile _was the strongest multivariable predictor in each of the best overall models of MACE, all-cause mortality, and non-fatal events. For MACE prediction, LAEF_Contractile_, history of cardiac bypass surgery, and left bundle branch block on ECG were selected to form the best overall model. Figure 3 presents the model LRχ^2 ^of the selected multivariate variables in each of the best overall models for MACE, all-cause mortality, and non-fatal events, respectively (Figure 3A-C). In the second multivariable approach, adjusted to the patients' age, gender, and LV ejection fraction, LAEF_Contractile _maintained strong and independent prognostic association with MACE (model LRχ^2 ^increased from 4.99 to 22.02, P < 0.0001), all-cause mortality (model LRχ^2 ^increased from 4.54 to 15.88, P < 0.0001), and non-fatal events (model LRχ^2 ^increased from 5.05 to 10.58, P = 0.005).

### Myocardial Ischemia and Left Atrial Mechanical Function

One hundred and six patients (50%) of the cohort underwent CMR for evaluation of myocardial ischemia. Among them 52 (49%) underwent dobutamine stress cine and 54 (51%) underwent adenosine stress perfusion imaging. Nineteen (17%) of the 106 patients were found to have myocardial ischemia during stress CMR. Twenty-six (25%) of the 106 patients experienced MACE during study follow-up, among them 11 died. Mean LAEF_Contractile _was not significantly different between patients with or without myocardial ischemia (33 ± 11 vs. 35 ± 11, P = 0.38). In the subgroup of patients who underwent CMR ischemia evaluation, presence of ischemia did not demonstrate any significant association with MACE, all-cause mortality, and non-fatal events (model LRχ^2 ^0.90, 0.08, and 0,07, respectively). When presence of myocardial ischemia was entered into a model that contains LAEF_Contractile_, the significant associations of LAEF_Contractile _with MACE and all-cause mortality, respectively, were not altered (hazard ratios of LAEF_Contractile _adjusted to ischemia, 1.97 and 2.17; P = 0.0009 and 0.004, respectively).

## Discussion

In this present study of hypertensive patients without significant valvular disease or myocardial infarction, a decreased contribution of LA contractile function to ventricular filling during diastole was strongly predictive of MACE, mortality, and non-fatal events. We found that in these patients, proportional contribution of the LA contraction as measured by the LA Contractile/Total ratio are strongly associated with MACE and patient mortality. These observations were true even after adjustment for age, gender, LA volume and left ventricular ejection fraction. This postulates that altered LA mechanical contractile function by CMR in patients with or at risk for diastolic dysfunction from hypertension provides unique and independent prognostic value to adverse cardiovascular outcomes. To our knowledge, this is the first study which demonstrates a relationship between altered LA contractile function and CV outcomes, including mortality, in hypertensive patients.

In normal humans, passive emptying of the LA (a combination of the reservoir and conduit functions of the LA) during ventricular diastole contributes 75-80% of LV filling. LA contractile function normally contributes approximately < 25% of the total LV filling volume[19], but becomes increasing important to LV diastolic filling as ventricular stiffness progresses. In the mild stage of impaired LV relaxation, the LA contractile function increases and can contribute up to 38% of LV filling[1]. However, as LV stiffness progresses to moderate or severe range with marked increases in LV filling pressures, the contribution of LA contraction falls below the normal contribution, and the LA becomes mainly a passive conduit for ventricular filling [3,10]. Given what is known about the physiology of LA mechanical function, the present findings would seem to suggest that the increased risk of MACE and mortality might be conferred mostly on those patients with more severe (i.e. grade III or IV) restrictive LV diastolic dysfunction and thus decreased LA contractile function. Indeed, although patients in the general population with all grades of diastolic function are at increased risk of adverse CV outcomes and mortality, the risk is most pronounced in those patients with restrictive (grade III or IV) LV diastolic function [20]. This is also consistent with the known limited ability of the LA to reflect changes in mild or moderate LV diastolic dysfunction. Pritchett et al. has previously reported that in the general population, indexed LA volume has the highest sensitivity and specificity for the detection of severe (grade III or IV) LV diastolic dysfunction [21]. The same may be true of LA contractile function.

In this study, CMR is able to quantify LA contractile function at high spatial resolution for border delineation and adequate temporal resolution to capture LA motion throughout the cardiac cycle. We demonstrated such LA quantitative results are highly reproducible. While echocardiographic studies have assessed the relationship between LA volume and clinical outcomes, the prognostic relationship between the different components of LA mechanical function and clinical outcomes has not been well studied. We postulate that exposure of the LA myocardium to elevated left ventricular diastolic pressure can result in LA myopathy which can be quantified by LAEF_Contractile _utilizing CMR as in the current study. Conformed to the existing body of echocardiographic literature, we used planar images of the LA acquired along its long axis and then used two dimensional measurements and standardized formulas to calculate LA volumes by the biplane area length method. It is therefore conceivable that the CMR findings of our study can be applied to similar patients undergoing echocardiographic assessment.

LA size has long been reported to be a sensitive and load-independent marker of both the severity and duration of LV diastolic dysfunction[22-24]. There is substantial and growing evidence pointing to the clear prognostic value of LA size in selected clinical populations. In the general population, increased LA volume has been shown to predict atrial fibrillation [23-28] stroke [22,29], and incidence of heart failure [30,31]. In addition, several authors have demonstrated the role of increased LA volume in predicting mortality in populations with pre-existing cardiovascular diseases, such as post-myocardial infarction or with LV dysfunction [32-35]. We believe that this study adds to this knowledge by showing that in patients with chronic hypertension, LA contractile function represents an additional prognostic tool by characterizing the burden of underlying diastolic dysfunction not assessed by current imaging techniques. Moreover, this study demonstrates the feasibility of using CMR to assess the LA in the clinical setting to predict cardiovascular outcomes.

### Limitations

There are a number of study limitations. This study was a retrospective referral-based cohort study at a single center. All of the subjects studied had been referred for CMR examination, while the clinical indications of such were appropriate, the generalizability to other populations with hypertension may be limited. Only 50% of the current study cohort underwent stress CMR evaluation for myocardial ischemia. While we found that presence of myocardial ischemia did not seem to alter the prognostic significance of LA contractile function with the clinical outcomes, the current report does not have the study design or power to properly address whether myocardial ischemia could have been a confounding variable. The method of LA volume quantitation in this study can only provide estimates of LA volumes since it has not been validated against the axial stacked volumetric imaging. The study is also limited by its sample size and the small number of patients who experienced MACE, therefore some degree of model overfitting in the multivariable analyses may have occurred. For this reason, confirmation of the current study findings and exploration of potential therapeutic implications that can alter LA mechanics are necessary in a larger prospective study. Finally, concurrent load-independent measures of LV diastolic function such as tissue Doppler velocity are not available in the current study, thus prohibiting probing in the mechanism of the significant prognostic findings being reported in the current study. Such association of LA contractile function with imaging markers of diastolic dysfunction and serum markers such as naturetic peptide levels[36], will need to be studied prospectively.

## Conclusions

In patients with a history of chronic hypertension, a decreased contribution of LA contractile function to ventricular filling during diastole appears to be a novel predictor of adverse cardiac events and death.

## Competing interests

The authors declare that they have no competing interests.

## Authors' contributions

MK performed and MJH helped in post-processing and off-line analyses of the CMR images. MK, and KS collected clinical follow-up data in all of the patients. ST and TH participated in the design of the study, assisted in data entry, and performed the statistical analysis. RK conceived of the study, participated in its design, oversaw data acquisition at all levels, and helped to draft and revise the manuscript. All authors read and approved the final manuscript.
